# Pluripotent Stem Cells for the Treatment of Retinal Degeneration: Current Strategies and Future Directions

**DOI:** 10.3389/fcell.2020.00743

**Published:** 2020-08-14

**Authors:** Larissa Ikelle, Muayyad R. Al-Ubaidi, Muna I. Naash

**Affiliations:** Department of Biomedical Engineering, University of Houston, Houston, TX, United States

**Keywords:** stem cell, pluripotent, IPSCs, retinal regeneration, embryonic stem cell

## Abstract

Stem cells have been part of the biomedical landscape since the early 1960s. However, the translation of stem cells to effective therapeutics have met significant challenges, especially for retinal diseases. The retina is a delicate and complex architecture of interconnected cells that are steadfastly interdependent. Degenerative mechanisms caused by acquired or inherited diseases disrupt this interconnectivity, devastating the retina and causing severe vision loss in many patients. Consequently, retinal differentiation of exogenous and endogenous stem cells is currently being explored as replacement therapies in the debilitating diseases. In this review, we will examine the mechanisms involved in exogenous stem cells differentiation and the challenges of effective integration to the host retina. Furthermore, we will explore the current advancements in *trans*-differentiation of endogenous stem cells, primarily Müller glia.

## Introduction

Amongst vertebrates, the retina is a highly conserved tissue (Mitashov, 2004). However, unlike zebrafish and many amphibian species, the singular inability to regenerate damaged tissue in the mammalian retina has drastic ramifications. Retinal diseases or visual impairment inflict over 237 million people worldwide and may impact more than 587 million by 2050 (Bourne et al., 2017). Therefore, optimizing stem cell integration and developing strategies that encourage retinal regeneration are critical for preempting this massive rise in retinal diseases in the near future.

Cellular potency is defined as a cell’s ability to give rise to different cell types (Binder et al., 2009). Pluripotent cells under standard conditions will continue to propagate, producing daughter cells that are identical to the mother cell (Kolios and Moodley, 2013). However, under developing or inductive conditions, pluripotent cells can escape the replicative cycle, and produce differentiated cells, completely dissimilar to its progenitor (Kolios and Moodley, 2013).

Embryonic, induced, and reprogrammed stem cells are pluripotent cells currently being explored for retinal cell replacement therapies. Embryonic stem cells are derived from the blastocyst of a developing embryo and can generate cells of the three germ layers – the mesoderm, endoderm, and ectoderm (Vazin and Freed, 2010). Contrarily, induced pluripotent stem cells originate from adult somatic cells and are reprogrammed by the transfection of critical transcription factors, such as SOX2, Klf4, c-Myc, and Oct4. Once expressed, these factors induce the transformation of adult somatic cells into cells of the germ layers and can be re-differentiated into any desired cell type (Takahashi and Yamanaka, 2006). Reprogramming of endogenous cells is another viable method of stem cell generation (Jeon and Oh, 2015). The adult eye is actually comprised of cells with dormant potency (Bernardos et al., 2007). These cells include Müller glia (Turner and Cepko, 1987; Bernardos et al., 2007) and cells derived from the ciliary pigment epithelia (Ballios et al., 2012; Jeon and Oh, 2015). As these cells possess neurogenic genes, amplification of specific pathway mediators can induce regenerative pathways (Hamon et al., 2019), mobilizing these cells to replace damaged tissue (Yao et al., 2018).

Historically, treatment for degenerative diseases of the retina are marginally effective (Hamel, 2006; Ambati and Fowler, 2012). In late stage retinopathies and inherited malignancies, extensive cellular dysfunction and oxidative stress ultimately lead to cell death (Wert et al., 2014), structural damage, neuronal rewiring (Marc and Jones, 2003; Jones and Marc, 2005) and vision loss. Vision loss is an incredible challenge for treatment, especially in the post-mitotic environment of the retina. Additionally, retinal diseases, such as retinitis pigmentosa (RP), tend to be heterogenic and highly varied, even in the event of mutations on the same locus (Wert et al., 2014; Verbakel et al., 2018). Clinical trials in molecular therapies such as gene and protein therapies are currently underway and have some promising results, especially for patients with defects in the retinal pigment epithelium (RPE) (Smith et al., 2009). The FDA approved Luxturna^TM^ (voretigene neparvovec-rzyl) for gene therapy for RP and Leber’s Congenital Amaurosis (LCA) caused by mutations in retinoid isomerohydrolase (RPE65) (Weng, 2019). But for the most part, gene therapy is ineffective when the majority of the cell population is absent after end stage degeneration (He et al., 2014). Furthermore, individual therapies will have to be developed to address each disease. Therefore, exploring the mechanism of exogenously and endogenously generated pluripotent stem cells is critical for the treatment of retinal degenerative diseases. In this review, we will explore the advancements made in the generation of exogenous pluripotent stem cells and their translation to viable replacement therapies. Additionally, we will present the progress of elucidating the regenerative mechanisms involved in activating regenerative pathways in endogenous stem cells of the retina.

## Environment of the Degenerative Retina

Certain properties of the retina make it highly amenable to stem-cell therapies. The retina is self-contained, isolated from critical tissues that may contribute to treatment-related systemic effects, and the RPE and tight vasculature of the retina create an immune-privileged environment (Eveleth, 2013; Ramsden et al., 2013). Therefore, delivery and implantation are not the most prohibitive challenges to stem cell therapy; instead the most difficult obstacles are cellular survival and integration into the cellular network of the host retina (Wong et al., 2011; He et al., 2014), which are dependent outcomes. So, in order to properly address the advancements in stem cell retinal therapy, our exploration will begin with a discussion of the retinal environment before and after the onset of disease.

Briefly, the retina is a highly specialized stratified tissue, each layer containing constituents integral for light capture and signal propagation. The RPE is a monolayer of polarized epithelial cells that forms the primary layer and is critical for supporting photoreceptor cells (Strauss, 2005). The cells of the RPE have a host of functions not limited to, retinoid recycling, nutrient delivery, and light absorption (Strauss, 2005). The primary sensory cells of the retina are the photoreceptors (PRs), which sit directly below the RPE, but within its microvilli. PRs are primary neurons with specialized cilia that house the chromophore necessary for visual transduction. Photoreceptors can be either rods or cones, which have significant structural, functional and molecular differences. The most consequential difference, however, is the chromophore. The cone chromophores are important for colored vision, while rods are more sensitive and are responsive in low light conditions (Kawamura and Tachibanaki, 2008). Once the chromophore has been activated by light, an electro-chemical signal is propagated to the synaptic terminal which activates second order neurons. Horizontal, bipolar, and ganglion cells, in that order, integrate and transmit the signal to the brain, were it is finally perceived as vision (Remington, 2012). Müller glia, astrocytes, and microglia surround both primary and second order neurons (Remington, 2012), and are important for maintaining retinal homeostasis.

Consequently, any disruption to this delicate architecture can lead to deleterious changes to the retinal environment. RP and age-related macular degeneration (AMD) are emblematic retinal atrophic pathologies that highlight the major themes of retinal degeneration and are ideal candidates for stem cell therapies.

### Retinitis Pigmentosa

Retinitis pigmentosa is the most common form of inherited retinal diseases (Wert et al., 2014) whereby about 190 genes have been identified that are directly linked to the disease (Daiger et al., 2013). However, despite its etiological diversity, disease pathogenesis is similar between patients (Wert et al., 2014) and can be used to highlight the changes to the retinal environment as the disease progresses. In early stages of RP, patients may experience night blindness, which is indicative of rod photoreceptor dysfunction or loss. Mutant genes associated with RP disrupt proteins involved in photo-transduction or proteins integral to photoreceptor structure (Ferrari et al., 2011). As a result, the continuous insult of gain or loss of function mutations within photoreceptor proteins ultimately induces apoptosis of the cell (Wert et al., 2014). Generally, photoreceptors loss begins in the periphery, and slowly progresses to the mid-periphery and center of the retina. At this late stage, patients may report “tunnel vision” where sight is predominately attenuated at the periphery, and central vision is all that remains (Wert et al., 2014). Depending on the mutation, rod loss is proceeded by the loss of cone photoreceptors, causing complete vision loss (Verbakel et al., 2018).

Cone photoreceptor degeneration is the most debilitating aspect of RP. Though many mutations are rod-specific, death of cones generally ensues (Wert et al., 2014). Many suggest that rods provide critical trophic components into the retinal environment that ensure cone survival (Kelley et al., 2017) and because rods are the major cellular component of the photoreceptor layer, they contribute essential infrastructure for the cones (Steinberg, 1994; Ripps, 2002). Nevertheless, RP has demonstrated that cone survival is contingent upon rod photoreceptor health. As photoreceptors disappear, RPE cell death follows. RPE cells are highly myelinated and the deposition of melanin is a characteristic feature observed in the fundoscopy of RP patients (Wert et al., 2014).

Many RPs are monogenic (Weleber, 2005), which highlights the broad interconnectivity of the retina. One mutation can cause a cascade of negative events, eventually rendering most of the tissue completely dysfunctional. Dysregulation and degeneration of RPE cells as a consequence of mutations in photoreceptors is an example of one of these secondary effects (Stuck et al., 2014). These effects extend to the choroidal and inner retinal vasculature, which are markedly attenuated in RP patients (Yang et al., 2018). Under normal conditions the oxygen levels in the retinal choroid are near arterial levels (Yang et al., 2018). In the degenerative environment of RP, the oxygen levels are even higher as oxygen consumption is reduced as a result of reduced photoreceptors (Yang et al., 2018). Vascular changes reduce nutrient bioavailability which may further perpetuate RPE and photoreceptor dysfunction. These drastic changes to metabolism and homeostasis encourages the recruitment of glial cells to the outer retina and the secretion of tumor necrosis factor alpha (TNF-α) (Roesch et al., 2012) and other proinflammatory factors from Müller glia.

Mutations in rhodopsin form the majority of autosomal dominant RP cases in North America and serve as an example of monogenic RPs (Mendes et al., 2005). Different mutations within the gene can cause a host of varying dominant negative and gain-of-function phenotypes. For instance, class I mutations do not affect the tertiary structure of rhodopsin, but cause mislocalization of the protein. Class II mutations cause misfolding of the protein, and the large majority of these aberrant proteins aggregate in the cytosol and ER, generating a cytotoxic environment (Mendes et al., 2005). Irrespective of the mutation, over time photoreceptor and retinal atrophy significantly attenuates function, damages the cellular infrastructure, and the inner retina undergoes significant remodeling to accommodate these drastic changes (discussed in a subsequent section).

Because the pathogenesis of RP is multifactorial, many therapies have been explored to improve prognoses, however, many can only slow disease progression and are not curative. Therapy with vitamin A and DHA has a short-term positive effect, but effective dosage of vitamin A were approximately 15,000 units/day, which can be toxic (Sahni et al., 2011). Gene therapy is a strategy that has had promising results, but still has major challenges considering the vast number of different genes involved in RP, the diversity of clinical presentations, and the difficulties in transfecting PRs (Smith et al., 2009).

Neuroprotection involves the use of trophic factors such as ciliary neurotrophic factor (CNTF) or brain derived neurotrophic factor (BDNF) to enhance cell survival and curb the effect of pro-apoptotic factors (Sahni et al., 2011). Encapsulated cells secreting CNTF were delivered to the vitreous in degenerative animal models and preservation of retinal structure was observed. This modality of trophic delivery to the retina advanced to clinical trials. However, the large majority of trophic therapies are in very nascent stages since these factors have a short half-life requiring frequent applications through invasive means. Although beyond the scope of this review, stem cells can also be used to elicit this neurotrophic effect (Xu and Xu, 2011). Recently, intravitreal injection of mesenchymal cells altered to overexpress BDNF has been shown to attenuate apoptosis in RD6 mice (Lejkowska et al., 2019), demonstrating the versatility of stem cell approaches.

### Age Related Macular Degeneration

Analogous to RP, age related macular degeneration (AMD) has a multi-faceted etiology and devastates the aging population. Recent indications suggest that by 2040 over a quarter of a billion people worldwide may be affected by AMD (Wong et al., 2014). AMD is marked by chronic malignant changes to structures that support the macula, such as the choroid, RPE, and Bruch’s membrane. As structures are compromised, patients report loss of central vision (Zając-Pytrus et al., 2015).

Based upon symptom onset and clinical presentation, AMD is divided into early and late stage AMD (García-Layana et al., 2017). Initially, before patients experience any disruption to vision, the Bruch’s membrane will start to thicken due to lipid-protein deposits (Young, 1987). These deposits may occur under normal conditions, however, when these deposits aggregate to approximately 125 μm or larger they become pathogenic (The Eye Diseases Prevalence Research Group, 2004). These deposits (aka drusen) inhibit proper flux and communication between the Bruch’s membrane and the RPE (The Eye Diseases Prevalence Research Group, 2004). Like the inner retina, the RPE serves as an integration junction, responding to stimuli from the outer retina, choroid, and Bruch’s membrane. The deposits critically injure the RPE and debilitate its important functions. Without the proper flux of nutrients and environmental signals, the RPE is now susceptible to a host of injurious events, such as oxidative stress, metabolic dysfunction, reduced photoreceptor interconnectivity, and inflammation (Young, 1987).

In fact, inflammation plays an important role in AMD pathology (Akhtar-Schäfer et al., 2018). Previous studies have indicated that over reactive immune responses by the innate immune system of the retina (Ambati et al., 2013) and slow chronic infiltration of inflammatory immune components promote degeneration of the RPE and photoreceptors. As previously mentioned, the RPE is part of an intricate architecture that imparts immune privilege to the outer retina (Sugita, 2009). However, in cases of AMD, where autoimmunity, aberrations in microglia behavior (Ambati et al., 2013), and RPE degradation allow for dendritic and macrophagic invasion (Ambati et al., 2013), the mechanisms that sequester the retina from circulating immune elements break down creating a compromised microenvironment (Ambati et al., 2013), further complicating treatment, as immune privilege is an aspect of the retina that makes it highly amenable to stem cell therapies.

Nevertheless, photoreceptor atrophy, specifically in the macula, is the worst eventuality (Young, 1987). In 10% of cases (Halpern et al., 2006), this RPE dysfunction can cause the upregulation of pro-angiogenic factors like vascular endothelial growth factor (VEGF) (Hernández-Zimbrón et al., 2018). The promotion of angiogenesis in this already fragile environment will ultimately cause blindness when left untreated. Angiogenic presentation in AMD, is called “exudative” or “wet” AMD (Hernández-Zimbrón et al., 2018). VEGF causes neovascularization in the choroidal strata. As the oxygen demand is highest at the macula, most of this neovascular invasion may occur at the macula. The newly formed vascular infrastructure is weak and immature, causing excessive leakage which reaches the macula, causing detachment and RPE and PR atrophy and ultimately blindness (Hernández-Zimbrón et al., 2018).

Mitigating degeneration in AMD is challenging as genomics have unearthed many potential genes and mechanisms that may contribute to its pathogenesis (Fritsche et al., 2016; Yan et al., 2018). By improving diet, discontinuing smoking, and reducing exposure to environmental risk factors, patients can slow disease progression (Khan et al., 2006; Moutray and Chakravarthy, 2011). Additionally, photocoagulation laser therapy had been previously implemented to prevent neovascular damage, but relapses were not uncommon (Moutray and Chakravarthy, 2011). More efficacious treatments are VEGF inhibitors, such as ranibizumab which is a monoclonal antibody binding to VEGF-A (Moutray and Chakravarthy, 2011). Most recently, a better FDA approved drug, Aflibercept, has become a common treatment of wet AMD. It is a fusion protein that functions similarly to ranibizumab or bevacizumab, only with improved inhibition of VEGF and requires less frequent dosing (Sarwar et al., 2016). VEGF inhibition, however, is only effective in a third of patients (Moutray and Chakravarthy, 2011), thus additional investigations are exploring therapies that address pro-angiogenic factors upstream and downstream of VEGF (Moutray and Chakravarthy, 2011). Despite partial successes with wet AMD, there are still no curative treatments for dry AMD (Zając-Pytrus et al., 2015).

### Retinal Remodeling

**FIGURE 1:**
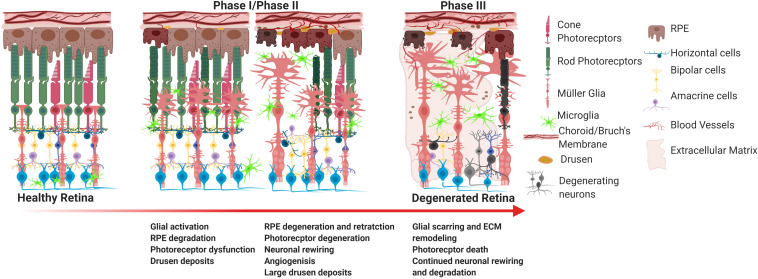
Progression of retinal degeneration. The major hallmarks of degeneration include RPE degradation and photoreceptor atrophy. In the case of wet AMD and some forms of RP, choroidal neovascularization may occur. In the last phase of degeneration, the large majority of the subretinal space undergoes drastic glial remodeling and scarring, as represented in phase III, which significantly obstructs cellular integration of stem cells.

As previously mentioned, integration of transplanted cells into the host retina remains a substantial challenge for stem cell therapy. The retina is a dynamic system that undergoes histological, molecular, and genetic remodeling to accommodate the degenerative environment under pathogenic conditions as illustrated in Figure 1. As photoreceptors apoptose, the inner retina loses sensory input (deafferentation) which is the primary reason for remodeling (Jones et al., 2012). Remodeling is composed of three phases. Phase I is characterized by photoreceptor dysfunction and stress which is the result of the initial pathological insult. In phase II photoreceptor apoptosis encourages glial migration, remodeling in the outer retina, and morphological changes to second order neurons. Müller cells and astrocytes leave behind a glial seal between the remaining RPE and neural retina. Phase III is a period of constitutive neural rewiring, and glial and vascular remodeling. These phases are highly variable as degeneration varies between diseases and pathogenesis and rate of decline are dictated by disease mechanism and the individual patient. For example, RPs with delayed cone degeneration can extend the onset of phase III.

Many of the changes that happen in phase I occur on a molecular level and do not manifest clinically (Jones et al., 2012). Contrarily, phase II and phase III are quite dynamic. As a consequence of photoreceptor apoptosis, bipolar cells can exhibit either telodendria or dendritic retraction (Jones et al., 2012). Investigations using mouse models, have determined that rod bipolar cells are the first to demonstrate significant structural changes, followed by ON bipolar cone cells, and finally OFF bipolar cells (Strettoi, 2015). Because bipolar cells express metabotropic glutamate receptor (mGluR6) and undergo glutamatergic synapses with photoreceptors, these cells are the first to experience the effects of deafferentation as photoreceptors die (Strettoi, 2015). Bipolar cells can rearrange and migrate forming aberrant connections and ectopic synapses (Strettoi, 2015). Continued photoreceptor atrophy causes hypertrophic glial cells to migrate to the outer retina and begin gliotic remodeling (Jones and Marc, 2005). They digest dying cells, remodel the subretinal space, and rearrange molecular components of the extracellular space to develop a gliotic “seal” (Marc and Jones, 2003). Large amounts of chondroitin sulfate proteoglycans are deposited into retinal extracellular matrix (ECM) by glial cells, which inhibits axonal growth (Fawcett, 2009). Horizontal cells and amacrine cells undergo slower remodeling exhibiting the same axonal sprouting or retraction that may be observed by bipolar cells (Jones et al., 2012). Surprisingly, ganglion cells are the least affected by photoreceptor loss (Strettoi, 2015) whereby major aberrations are only seen long after outer retinal disintegration (Strettoi, 2015).

As the pathophysiological changes in the retina occur, the inner retinal remodeling can be overlooked, as photoreceptor degeneration is the primary event. However, understanding of the topographical and molecular changes in the inner retina are critical to developing the most efficacious stem cell therapeutics. This remodeling timeline must be carefully considered, to ensure that connection between bipolar cells can be re-established and extensive gliosis has not occurred. Nevertheless, in order to advance transplantation of exogenous stem cells, which for the most part have only been marginally successful, therapies should consider the amelioration to the degenerative environment in conjunction with the delivery of stem cells.

## Exogenous Stem Cells

### Embryonic Stem Cells and Retinal Differentiation of Pluripotent Cells

Embryonic stem cells (ESCs) are pluripotent cells that originate from the blastocysts of a developing embryo (Itskovitz-Eldor et al., 2000). Their pluripotent capacity has been extensively examined in mouse and in *in vitro* models (Itskovitz-Eldor et al., 2000), and it is this potent ability that has garnered excitement for the development of therapies for a wide range of neurodegenerative diseases. However, in order to generate a viable therapeutic, ideal inductive conditions must exist for ESCs to differentiate and acquire attributes of retinal cells.

To establish induction methodology, it was important to identify critical factors of retinal development which may encourage ESC differentiation. Previous data from chick retina has elucidated the role of WNT signaling in neural determination (Cho and Cepko, 2006; Lamba et al., 2006). Identified sequential steps of retinal development which were determined to be sufficient for ESC induction. In order to begin what was coined by Lamba “retinal determination,” human embryonic stem cell (hESC) aggregates were initially cultured with Dickkopf-1 (dkk1), noggin, and insulin-like growth factor-1 (IGF-1) (Lamba et al., 2006). Dkk1 is a WNT/β catenin antagonist, and noggin inhibits the bone morphogenic pathway (BMP) (Lamba et al., 2006). IGF-1 was identified to promote ocular development in *Xenopus* embryos (Pera et al., 2001). Eye field associated transcription factors (e.g., Paired box protein and PAX6) were identified in ESCs after 3 weeks, and after longer exposure to inductive media, analysis indicated that the large majority of cells had ganglion, horizontal and amacrine cell characteristics. Co-culturing with retinal explant encouraged the population of ESCs to develop rudimentary photoreceptor attributes, as a small population of cells expressed recoverin (Lamba et al., 2006).

However, requiring retinal explant for photoreceptor differentiation would significantly limit the use of ESCs for clinical applications. Osakada et al. (2008) developed a strategy that implemented γ-secretase inhibitor, DAPT, to effect Notch signaling pathways and Left-Right Determination Factor A (Lefty A) to inhibit WNT signaling. By using these inhibitors, Osakada et al. (2008) produced a small population of cone rod homeobox positive (CRX^+^) cells, significantly more than in the absence of DAPT. CRX^+^ positive cells do not only indicate photoreceptor precursor cells, but they also suggest post-mitoses. Furthermore, step-wise treatment with taurine, retinoic acid, Sonic hedgehog (Shh), and fibroblast growth factor (FGF), continued to push ESCs toward a photoreceptor-like cell as they expressed rhodopsin and recoverin following exposure to these morphogens.

Both the work of Osakada and Lamba created a framework in which to induce retinal cells from embryonic stem cells and further validated that the inhibition of BMP and WNT pathways is critical for retinal determination. In the developing optic cup, centralized progenitor cells transform to become the different retinal cell types, while progenitor cells at the periphery become non-neural cells, i.e., the ciliary body and iris (Cho and Cepko, 2006). Since WNT signaling is integral to these determinations, upregulation of WNT activity is indicative of a non-neural fate, while inhibition of WNT may encourage differentiation to neural cells. In the developing retina SOX2 plays a modulatory role restricting WNT activity (Heavner et al., 2014).

In addition to retinal determination, the combined inhibition of WNT and BMP pathways can be modulated to control rod or cone photoreceptor lineages. Both noggin and chordin are antagonists to BMP and are frequently used in retinal determination of IPSCs and ESCs (Smith and Harland, 1992; Messina et al., 2014). BMP, in a neural context, is important for glial cell development (Ueki et al., 2015b). Inhibition of WNT and BMP may yield 12% CRX expressing cells (Lamba et al., 2006), while only 4% of the cells may express rod photoreceptor markers, and 0.01% may express cone photoreceptors proteins (Lamba et al., 2009). Zhou et al. (2015) reported that the use of Coco (a factor from the Cerberus family), as an effective WNT and BMP inhibitor, not only promoted photoreceptor neurogenesis in hESCs, but also increased the propensity for cone photoreceptors, up to 60%. These cone photoreceptors were transplanted into 2-day old pups, whereby they were able to differentiate and integrate into the host retina and exhibit similar morphology to the endogenous photoreceptors (Zhou et al., 2015). The use of Coco for cone differentiation presents a viable method for developing cone photoreceptors cell replacement therapies in RP and macular degeneration.

The aforementioned strategies, however, generated retinal precursor cells in substantially low yields, over an extensive period of time, up to 120 days in some instances (Mellough et al., 2012). Proceeding studies have ventured to improve upon this groundwork by ameliorating culture conditions. Yields demonstrated considerable improvement by simply controlling the size and population of embryoid bodies. By limiting the size to approximately 200 μm, the total population of CRX^+^ cells was 78%, and further increased to 93% by negative selection of hESCs (Yanai et al., 2013). Moreover, stepwise analysis of morphogens and media supplements has shortened the time frame for differentiation, and isolated specific supplements (such as B27) that encourage a neural or retinal fate more expeditiously (Mellough et al., 2012).

### Induced Pluripotent Cells and Retinal Differentiation

The use of embryonic stem cells, specifically human embryonic stem cells, comes with ethical and sourcing limitations. As a consequence, Takahashi and Yamanaka developed a method for inducing pluripotency in somatic cells by viral-vector mediate transfection using the four transcription factors previously mentioned (Takahashi and Yamanaka, 2006). After transfection of these factors, cells from the three germ layers should be present (Takahashi and Yamanaka, 2006). Originally, established using adult mouse fibroblasts, advancement in the technology have indicated that virtually any somatic cell can be used, and as they can be patient-derived, once differentiated, these cells appear to show no or limited immune rejection (Mandai et al., 2017). Transplanted RPE sheets of autologous induced pluripotent stem cells (IPSCs) were tested in patients with wet AMD and demonstrated no immune rejection a year after transplantation (Mandai et al., 2017).

The differentiation systems discussed in the prior section primarily employed serum-free embryoid body like (SFEB) culture conditions, where cellular aggregates are grown in serum free media for the preliminary stages of differentiation followed by a transfer to coated plates for attachment (Lamba et al., 2006). The resultant cells express photoreceptor specific markers and demonstrate reduced expression of mitotic proteins, suggesting the presence of photoreceptor precursors (Lamba et al., 2006). However, many of the cells do not possess the specialized cilia of the photoreceptor, the photoreceptor outer segment, which is inherent to photoreceptor functionality. From developmental studies, it has been well established, that retinal development depends not only on transcription and trophic factors, but a host of spatial, environmental, and temporal cues, which cannot be entirely recapitulated in 2D culture (DiStefano et al., 2018). Consequently, many IPSC protocols now involve the generation of retinal organoids by growing IPSCs in a three-dimensional environment, to establish an ECM and multi-dimensional cell-cell interactions. These organoid cultures can even expand to accommodate a bioreactive system that can simulate oxygen exchanges, and vascular delivery of nutrients, and waste removal (DiStefano et al., 2018).

Successful retinal differentiation of IPSCs is contingent upon establishing a 3D microenvironment in addition to the proper sequential additions of morphogens (Zhong et al., 2014). At early differentiation stages of *in vitro* differentiation, as the eye field develops, IPSCs will organize themselves and migrate to form a rudimentary architecture (Zhong et al., 2014). The eye field will continue to develop and begin expressing the transcription factors visual system homeobox 2 (VSX2) and microphthalmia-associated transcription factor (MITF) which indicate a neural retina and RPE fate, respectively. Following expression of VSX2, cells committed for retinal formation, continue finessing retinal lamination and will also start to form a retinal cup with a pseudo-RPE attached. Once retinoic acid and taurine are added at this stage, neurogenesis continues with the formation and self-organizing lamination of Müller glia and ganglion cells preliminarily, followed by photoreceptors, horizontal cells, amacrine cells, and finally bipolar cells, which in part recapitulates cellular development *in vivo*. *In vitro* recapitulation of embryonic retinal neurogenesis is itself a successful feat, however, the formation of functional outer segments demonstrates the importance of accounting for the microenvironment when differentiating IPSCs. In the laminated organoid, photoreceptors express both rod and cone opsins and present with distal-end budding structures emanating from the outer retina, similar to what is observed in the developing outer segment. Patch clamp responses indicated that maturing photoreceptors in the organoid are light responsive (Zhong et al., 2014).

#### Induced Pluripotent Cells as Models of Retinal Pathologies

Considerable clinical barriers still remain for the use of IPSCs, such as fear of oncogenicity, lack of homogeneity in differentiated cell populations, genetic instability, and the massive resources mandated for generating safe and effective lines from individual patients (Singh et al., 2018). Despite these limitations, they have become vital research tools for modeling retinal disease pathology and developing personalized therapies, bearing in mind the difficulty of recapitulating diseases in rodent models. As an example, Yoshida et al. (2014) generated IPSCs from a patient’s skin punch that presented with retinitis pigmentosa caused by a heterozygous rhodopsin mutation, were a glutamic acid was substituted with a lysine (E181K). The skin fibroblasts were transfected with the indicated transcription factors, and the presence of each germ layer was determined by generating teratomas. The genetic construct of the mutagenic IPSCs were introduced into a healthy patient cell line to determine that the point mutation in rhodopsin was the singular cause of degeneration. After this validation, the same culture conditions developed by Lamba were implemented by Yoshida to induce retinal determination and rod photoreceptor differentiation. Analysis of these generated rod photoreceptor precursors confirmed disease pathogenesis; primarily, they determined that ER stress and autophagy contributed to photoreceptor death. Using rapamycin and a host of other drugs, patients derived rod photoreceptors precursors responded well to drug cocktails. These critical findings can be taken to the clinic for further assessment.

A successful IPSC model, as in the example above, is able to confirm the genetic cause of disease, recapitulate disease phenotype, elucidate cellular mechanisms of the disease, and ultimately validate pathogenesis by positively responding to interventions (Doss and Sachinidis, 2019). Another example involved the generation of 3D organoids from patients with frame shift mutations in the retinitis pigmentosa GTPase regulator gene (RPGR) which causes a form of autosomal dominant retinitis pigmentosa effecting cilia function and morphology (Deng et al., 2018). Thus far, animal models have not been able to categorically reflect the disease phenotype exhibited by patients, therefore these *in vitro* models are critical to understanding disease pathogenesis (Hong et al., 2001). 3D retinal organoids were generated from urinary cells of three diseased patients and three healthy donors (Deng et al., 2018). Cells differentiated and organized into retinal strata, as they would in the fetal retina and became electrophysiologically responsive. The diseased retina demonstrated a thin outer segment and inner segment layer, aberrant outer segment morphology and rhodopsin transport, and mislocalization of opsins, providing significant insight into the causes of photoreceptor loss exhibited by patients.

To correct the frame shift mutations, the highly effective bacterial gene editing system, clustered regularly interspersed short palindromic repeats (CRISPR/Cas9), was implemented to repair the RPGR gene (Deng et al., 2018). Briefly, guided RNA bound to the Cas9 nuclease, excises the complementary sequence in the target DNA, where it can be replaced with the desired sequence (Baliou et al., 2018). Here, exon 14 of the RPGR gene was replaced with the healthy sequence (Deng et al., 2018). Resultant IPSCs were differentiated and showed significant biochemical and physiological improvement over the mutant IPSCs (Deng et al., 2018).

Therefore, genes with single mutations can be effectively modeled with IPSCs and treated with CRISPR gene editing technology or drugs and small compounds to target specific cellular mechanisms. However, limitations exist in the context of more complex polygenic diseases, as in the case of AMD or systemic diseases such as diabetic retinopathy. Disease can be the result of gene variants compounded with environment and systemic insults which cannot be recapitulated in the dish. So, rather than using IPSCs to model a disease of a single genetic etiology, Golestaneh et al. (2016) performed a study of IPSCs derived from patients of multiple genetic backgrounds and exposures, to provide an exhaustive analysis of AMD disease features and mechanisms. AMD patient samples with risk alleles for complement factor H (CFH), age-related maculopathy susceptibility 2/serine peptidase 1 (ARMS2/HTRA 1), LOC alleles, and complement factor B (FACTOR B) were used in this study. Physiological and biochemical assays indicated common features despite the different mutations, and a common underlying pathway (silent information regulator 1 (SIRT1)/peroxisome proliferator-activated receptor γ coactivator 1α (PGC-1 α) pathway) effecting mitochondrial biogenesis, which can be a potential therapeutic target that can be further explored using these RPE cells lines differentiated from patient samples.

### Transplantation and Factors Affecting Integration

As previously discussed, the degenerative environment is not amenable to cellular integration, so effective cell replacement therapy must overcome these environmental challenges. A host of characteristics must be considered before transplantation, such a degree of differentiation, number of cells, the cell types that may promote cellular health and integration, and whether the cells will be transplanted as a sheet or a suspension. Previously, there had been very few successful attempts of stem cell integration in the adult post-mitotic retina, not to mention, degenerative retinas (Young et al., 2000). In these cases, cells that do not integrate did not survive. Many observed a lack of long-term survival, eventual degeneration of the stem cell as result of chronic immune responses (Zhu et al., 2017), and in these advanced stages where a small number of endogenous cells remain, integration becomes a lot more difficult because islands of non-integrated cells coalesced and formed rosettes (Chacko et al., 2000).

In order to highlight the importance of the microenvironment to successful stem cell integration, Barber et al. (2013) conducted an experiment, in which stem cells were transplanted into six different genotypes with varying pathologies and rates of deterioration. In this experiment, age or extent of deterioration was accounted for. The phenotypes tested include Prph2^+/Δ307^, Prph2^rd2/rd2^, Rho^–/–^, PDE6β^rd1/rd1^, Crb1^rd8/rd8^, and Gnat1^–/–^, the first four being models of retinitis pigmentosa, the following, a model of LCA, and the latter a model of stationary night blindness. RP models and Gnat1^–/–^ showed integration similar to what was observed in wild type mice, where an average of 4,000 of 200,000 delivered cells were able to integrate into the host retina. The model of LCA demonstrated the greatest number of integrated cells, an average of approximately 10,000 cells. The rhodopsin null mice had the worst integration patterns, where almost no cells showed integration. This preliminary data suggested that even though the animals were injected at the same time point each disease had varying levels of degeneration at the time of transplantation which affected the success of integration.

Barber et al. (2013) also wanted to ascertain whether outer limiting membrane (OLM) integrity and gliosis had any effect on successful integration. In the Crb1^rd8/rd8^ model, where OLM integrity is already compromised, an interesting interplay between gliosis and OLM integrity was observed. It appears that the reduction in tight junctions in the OLM may actually improve stem cell integration as long as gliosis is relatively mild. At an intermediate time point where gliosis is not as rampant and OLM integrity is fully compromised, successful integration of stem cells in the Crb1^rd8/rd8^ was seen, even more integration than what was observed in samples 3 months younger. It is important to note, that the Crb1^rd8/rd8^ model had the largest number of integrated cells.

The rhodopsin null mice had the least number of integrated cells (Barber et al., 2013). To buttress the idea that modulating the microenvironment can improve integration, rhodopsin null mice were treated with siRNAs that target ZO-1 and disrupt OLM integrity. Chondroitinase was used to digest chondroitin sulfate proteoglycans which are inhibitory ECM molecules deposited as a result of glial scarring. With these modifications, transplantation was significantly more successful in this model, as well as in the wild type control, suggesting that simple transplantation may not be sufficient in highly degenerative models and that modulation of the microenvironment can significantly improve the success of stem cell integration and consequent long-term survival.

In addition to the microenvironment, dynamics of cell transplantation are also important aspect to consider. The degree of degeneration should dictate how cells are introduced into the environment whether as a cell suspension or as a retinal sheet. When there is still a remaining cohort of endogenous cells these pluripotent stem cells tend to fuse with the endogenous cells remaining (Decembrini et al., 2017). However, in advanced degenerative models, where most photoreceptors have died and the interphotoreceptor matrix and subretinal space are largely reduced, a more robust delivery system is required to supplement the highly disintegrated outer nuclear layer. Assawachananont et al. (2014) developed a retinal sheet to graft into PDE6β^rd1/rd1^ mice where most rods are absent by 3 weeks. Here, integration efficiency was tested at varying levels of stem cell differentiation and variation to sheet construction. What was observed was that younger, less differentiated cells that were not accompanied by a fully developed inner nuclear layer (INL), fared a lot better *in vivo* than the more highly differentiated organized sheets. These sheets, made of less differentiated cells, were able to form proper outer segments containing well aligned disk almost identical to wild type and were able to form synapses with host bipolar cells. In more differentiated retinal sheets, more rosette formation and less integration were observed. There was no significant difference between sheets developed from ESCs or IPSCs. Though this assessment did not include electrophysiological examination of the integrated stem cells, it provided important properties that can be taken forward to be implemented for the development of retinal sheets in advanced retinal degeneration.

Another feature the degenerative environment is the pro-inflammatory factors that are upregulated as result of the pathology (such as in the case of AMD) and the immunogenicity caused by transplantation itself (Nazari et al., 2015). A chronic immune response contributes to the lack of long-term survival of stem cells, especially with hESCs (Zhu et al., 2017). Immune suppressed mice where used to determine the effect of immunity on stem cell integration and longevity, using a compound animal that has a mutation in the CRX gene and is null for interleukin 2γ (IL2γ) (Crx^tvrm65^/*IL2r*γ^–/–^). This mutation in CRX recapitulates some of the visual symptoms described by patients with LCA. This investigation demonstrated that immunosuppression improved integration of hESCs; cells were able to mature, integrate with the existing INL, and were functional even after 9 months. Though the degree of integration was still less than the immunosuppressed wildtype control, it does indicate immune activity plays a role in long-term stem cell survival; additionally, as the number of integrated cells in the compounded animals was reduced from the wildtype (Zhu et al., 2017), it does imply that other elements also contribute to the death of grafted stem cells.

The reduced integration observed by Zhu et al. (2017) may attest to the influence of the inherent dysfunction of the retinal immune system under pathogenic conditions (Akhtar-Schäfer et al., 2018). As part of the retina’s immune privilege, circulating soluble elements such as macrophage inhibitory factors (MIFs) and transforming growth factor β2 (TGFβ2), and additional membranous components, play an important role in dampening and mitigating damage from the retina’s internal innate immune system or from other injury (Perez and Caspi, 2015). RPE cells, in particular, are integral to this homeostasis (Sugita et al., 2016). Specifically, they express complement components, minor histocompatibility molecules, anti-inflammatory cytokines (Holtkamp et al., 2001), and T-cell mediators and response elements (Mandai et al., 2017). In degenerative diseases, this ability to modulate immune activity becomes progressively dysfunctional and ineffectual as cellular atrophy worsens. Therefore, in addition to cell replacement, it is imperative that pluripotent stem cells retain the immunomodulatory properties of the cell type they become, in order to restore the immune regulatory mechanisms and prevent further insult to retinal tissues. IPSCs derived from healthy donors have demonstrated the ability to suppress T-cell activation, induce T-regulatory cells, and secrete immune suppressive factors such as MIF and TGFβ (Sugita et al., 2015). HESCs have also indicated a similar capacity, and appear to have a better immunomodulatory response in the absence of immune suppressive drugs, such as cyclosporine (Idelson et al., 2018).

As we will discuss in the following sections, allogenic transplantation using hESCs have made further strides to clinical translation than IPSCs for many reasons. One reason is that the polygenic and multifactorial nature of many retinal pathologies, make genetic corrections difficult, leaving allogenic transplantation from healthy donors the only viable option, currently. Nevertheless, genetic strategies to enhance immunomodulatory effects and reduce immunogenicity can significantly improve the disease microenvironment and stem cell survival, concurrently. Successful attempts using recombinant adeno-associated viruses (AAV) and CRISPR/Cas9 have yielded IPSCs with reduced immunogenicity by targeting human leukocyte antigen (HLA) polymorphisms, potentiating the availability of universal IPSCs for allogenic transplantation that will elicit no immune response (Gornalusse et al., 2017; Xu et al., 2019).

### Translation Capacities of IPSCs and ESCs and Current Clinical Trials

In order to meet clinical standards, many pluripotent stem cells should meet manufacturing standards that may pose challenging for some therapies. Propagation of stem cells may introduce genetic instability or phenotypic changes (Whiting et al., 2015). Moreover, though differentiation and induction occur under controlled settings, it is difficult to ensure 99% population homogeneity (a small sub-population may remain undifferentiated) (Whiting et al., 2015). These are just two of many requirement researchers must met before ESCs and IPSCs can be implemented in a clinical setting.

Nevertheless, a host of clinical trials using hESCs and IPSCs are underway for dry AMD, Stargardt’s disease and RP (Jayaram et al., 2014). One trial involves the subretinal transplantation of human derived RPE cells (OpRegen^®^) (NCT02286089, 2015). OpRegen had proven long term effectivity in Royal College of Surgeon (RSC) rat retina by significantly improving optokinetic thresholds and preventing further degeneration of the outer retina (McGill et al., 2017). The trial involved 24 participants 50 and older with dry AMD that presented without neovascularization (Banin et al., 2019). Interim reports indicate that the cells were well tolerated, and any adverse events were relatively mild (Banin et al., 2019). Most adverse events were post-operative problems and the formation of epiretinal membranes (Banin et al., 2019). However, some evidence suggested improvement in visual acuity as well (Banin et al., 2019).

In 2014, RIKEN undertook treatment of AMD using autologous IPSCs in two patients. The study was abruptly halted as three DNA deletions were identified in the male patient’s IPSCs. Though not proven to be tumorigenic, concerns regarding the deletions and the implementation of strict regulatory measures in Japan prevented further advancement of the study (Mandai et al., 2017) so a study using allogenic transplantation replaced it soon after (Garber, 2015). Recent changes to the IPSC induction cocktail and non-integrating episomal transfection strategies have been introduced, significantly diminishing worries concerning oncogenic potential of IPSCs. Consequently, NEI has entered phase I/IIa clinical trials for patients with dry AMD to test the safety and efficacy of autologous IPSC-derived RPE cells (NCT04339764, 2020). In 2012, 20 RP patients were recruited for phase I/IIa trials to determine the effectiveness of intravitreally administered bone marrow-derived stem cells (Reticell) (NCT01560715, 2011). Bone marrow-derived stem cells are adult stem cells, which are quite different from ESCs and extend beyond the scope of this review. However, patients reported improvement in the short term, but there was no long-term sustained improvement (Siqueira et al., 2015), suggesting use of stem cells in the ocular environment still have barriers to address.

## Endogenous Stem Cells

Thus far our exploration has examined the viability of pluripotent stem cells for cell replacement therapies in degenerative pathologies of the retina. However, the hostility of the degenerative environment and the low frequency of integration between graft and host remain prohibitive to clinical translation, as most of past and present clinical trials are focused on rescuing the RPE to preserve retinal health and not the photoreceptor. As a result, exploration of the retinal regenerative capacities has become another stem cell approach for addressing retinal pathologies.

The retina, for the most part, is entirely post-mitotic. Cells are terminally differentiated, and it has been shown that cell cycle reentry of photoreceptors may even induce apoptosis (al-Ubaidi et al., 1992). However, after analysis of injured retina in lower vertebrates, Müller glial and ciliary pigment epithelial cells have indicated some latent regenerative ability (Vihtelic and Hyde, 2000) and will be discussed in the subsequent sections.

### Müller Cells and *Trans*-Differentiation

In lower vertebrate, such as zebrafish, after acute injury, Müller glial will enter the cell cycle and produce retinal progenitor cells that can differentiate into any lineage. However, elucidating the molecular factors involved in this regenerative process has proven somewhat enigmatic. Following injury in zebrafish retina, reactive Müller cells (MC) dedifferentiate and produce neural progenitor cells (NPCs) (Pearson and Ali, 2018). This has an expansion effect, further recruiting more MCs and encouraging dedifferentiation (Pearson and Ali, 2018). NPCs increase in number, and migrate to the sight of injury, there they differentiate to the required cell type an reintegrate into the cellular network (Pearson and Ali, 2018). It is important to note, that microglia are essential to this MC response. When microglia were ablated in zebrafish using pharmacological agents, MCs had a delayed glial fibrillary acid protein (GFAP) response (indicating a delay in reactive gliosis) and the retina did not properly recover (Conedera et al., 2019).

What is the molecular basis for Müller cell *trans*-differentiation after injury? One hypothesis suggests a “negative-modulatory” model, where neural cell death may reduce the expression of an inhibitory factor that represses Müller regeneration, while another postulates that neural death may cause expression of a regenerative signal (Gorsuch and Hyde, 2014). Changes to expression levels of WNT signaling elements has provided some insight. After neural injury, WNT components are upregulated and inhibitors are drastically downregulated (Gorsuch and Hyde, 2014). It is interesting to note that WNT inhibition was critical to retinal differentiation in pluripotent retinal determination, indicating the modulation of WNT signaling is integral to retinal programming. In addition to WNT elements, TNF-α and TGFβ are important factors that mediate neurogenesis in zebrafish after injury (Sharma et al., 2020). TNF-α is released by the dying neural cells and is produced by Müller cells as well (Nelson et al., 2013), while TGFβ is released from the ECM by metalloproteases after injury. Both cytokines induce downstream expression of factors such as Achaete-scute homolog 1a (Ascl1a), Signal transducer and activator of transcription 3 (STAT3), and hairy-related 4 (her4.1), all of which are indispensable to NPC propagation from the Müller cell (Ueki et al., 2015a; Sharma et al., 2020). TGFβ also promotes cell-cycle exit of Müller cells after progenitor cell proliferation (Sharma et al., 2020). By understanding these factors, they can be implemented in mammalian retina to stimulate regeneration. Overexpression of Ascl1a in conjunction with deacetylase inhibitor allowed for Müller regeneration to INL neurons after injury in mouse retina (Ueki et al., 2015a). However, this was only successful after injury.

Under pathological conditions in the mouse retina, Müller cells reenter the cell cycle at G1 phase (Dyer and Cepko, 2000), but they do not replicate, instead reactive gliosis ensues. Nevertheless, this partial reentry indicates a latent regenerative ability which can be exploited. Yao et al. (2018) transfected Müller glial of adult mice with β-catenin under the control of a GFAP promotor using AAV (AAV; ShH10-GFAP-β-catenin) to promote cell cycle reentry (Yao et al., 2018). This injection was followed by another injection of OTX2, CRX, and NRL under the same promotor to stimulate rod photoreceptor regeneration. With this second injection another construct was delivered in order to express a reporter gene, td-tomato, under the control of the rhodopsin promotor. It was determined that Müller glial underwent one complete cell cycle before producing the daughter precursor cell. By examining td-tomato expression, the rod precursor initially had Müller-like morphology with radial processes. This cell produced two daughter cells, one of which maintained this Müller cell morphology and remained in the INL and eventually stopped expressing TD-tomato, as it became more Müller-like. While the other, Müller-derived rod-precursor, maintained td-tomato expression, was localized to the ONL, and matured into a rod photoreceptor that integrated into the cellular network of Gnat^rd17^Gnat^cpfl3^ mutants. Single cell recordings suggested presence of functional outer segments. This seminal work by Yao et al. (2018) provides strong evidence of the regenerative capacity of Müller glial using WNT signaling effector β-catenin. Given these findings, studies should employ a similar approach in highly degenerative mutants such as Rho^P23H^ or PDE6β^rd1/rd1^ where retinas undergo extensive remodeling and gliosis, then substantiate any recovery with more stringent physiological data.

Additionally, many studies have attempted to harness Hippo pathway effectors for Müller cell regeneration. The Hippo pathway is a highly conserved pathway necessary for mediating organ growth, and has been associated with hepatic regeneration (Grijalva et al., 2014). In order to inhibit organ growth in the mammalian cell, Hippo pathway effectors, Yes-associated protein (Yap) and Tafazzin (TAZ) undergo constitutive proteasomal degradation in the cytosol to prevent translocation to the nucleus where they would mediate the expression of mitogenic factors (Moya and Halder, 2019). Instead of inducing regeneration by gene transfer like Yao et al. (2018), Rueda et al. (2019) took a different approach by disrupting the mechanism inhibiting regeneration. Briefly, Rueda et al. (2019) demonstrated that by using an inducible transgenic model (Yap5SA), in which Yap is no longer degraded, cells can be reprogrammed to a progenitor-adjacent state. Once induced, a subset of cells (Yap5SA^+^ cells) were absent for GFAP, but significantly upregulated cyclin D1 and other cell cycle factors after retinal damage by *N*-methyl-D-aspartate (NMDA).

Thus far, investigations of Müller cell regeneration has yielded a superficial understanding of M ller cell response after injury, potentiating pathways to induce *trans*-differentiation or to block mechanisms that prevent regeneration in the mammalian retina. However, questions remain regarding the potential for regeneration during or after reactive gliosis. What changes? Does the retina have regenerative capacity long after the retinal environment has deteriorated? Current studies have validated the idea of retinal regeneration. Now, further evaluation of the regenerative capacity of the retina in severe degenerative models or models of end stage degeneration will move the field closer to clinical translation.

### Ciliary Pigment Epithelial Cells

The ciliary pigment epithelium (CPE) is an extension of the RPE and forms part of the ciliary body (Chang et al., 2016). Many have suggested that CPE cells may also have some regenerative capacity. In lower vertebrates the ciliary margin zone is a site of continued neurogenesis (Centanin et al., 2011), and the localization of the CPE stem cells suggests an analogous region of stem cells may also exist in the mammalian eye (Fischer and Reh, 2001; Raymond et al., 2006). Not only have there been clinical reports of neoplasm (though rare) forming in the ciliary body (Chang et al., 2016), but CPE cells have demonstrated a capacity for clonal expansion *in vitro* (using spheroid assay) where cells express retinal progenitor markers (nestin, CHX 10) and can *trans*-differentiate to neural cell types (Tropepe et al., 2000). Furthermore, cells at the ciliary margin of the mouse eye have shown relatively increased expression of cyclin D2, which is critical for G1/S phase transition (Marcucci et al., 2016).

Nevertheless, the stem cell population amongst these cells is quite small, and some even dispute this characterization of CPE cells as stem cells (Frøen et al., 2013). Gualdoni et al. (2010) demonstrated that CPEs were able to expand as a monolayer and as spheroids (to a lesser degree). Their expression profiles indicated the presence of progenitor markers, visual homeobox (CHX10) and retinal homeobox (RX), however, they never became stem cells because they concomitantly expressed and maintained epithelial characteristics. Additionally, exposure to the morphogens discussed in the prior section “Embryonic Stem Cells and Retinal Differentiation of Pluripotent Cells” did not induce CPEs to *trans*-differentiate and become photoreceptors precursors as was previously reported.

Many discrepancies exist regarding the regenerative capacity of CPE cells. RPE cells have also shown some capacity for self-renewal and can dedifferentiation to express progenitor markers. Transformed lines such hTERT-RPE1 can also be induced to express IRBP, recoverin, cone opsin, arrestin and transducin (Yan et al., 2013). Therefore, CPEs and RPE cells do appear to have multipotent capacities, and further research is required to understand the mechanisms involved in *trans*-differentiation. However, considering the small number of endogenous putative CPE stem cells in the mammalian retina, expansion and propagation is a prohibitive factor that may discourage the use of CPE cells for retinal regeneration.

## Concluding Remarks

**FIGURE 2:**
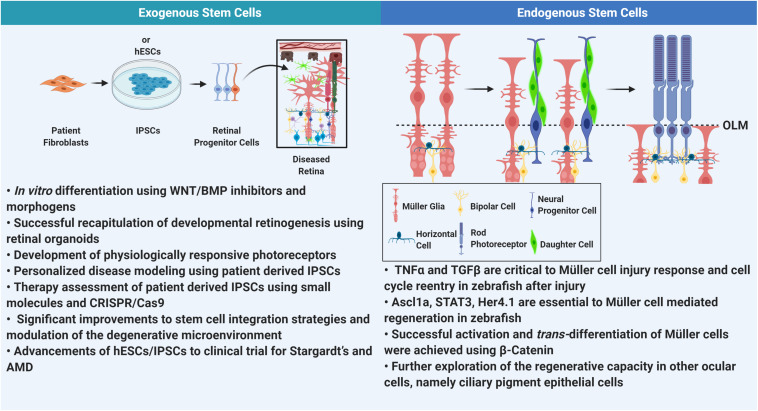
Comprehensive summary. In this review we address some of the major advancements in stem cell technology as it relates to retinal degeneration. Understanding the degenerative milieu has improved stem cell integration into the retina. However, thus far only RPE derived from hESCs and IPSCs have progressed to clinical trials. On the other hand, retinal regeneration in mammalian tissue is in very nascent stages. Elucidation of pathways involved in regeneration in zebrafish has been successful in isolating elements that can be used to mobilizing Müller glia for regeneration in mammalian retina. (OLM, outer limiting membrane).

Figure 2 highlights the major themes addressed in this review. However, considering these advances, RPE cells derived from hESCs are the first to show a strong translational capacity, as therapies are currently under clinical trials. However, ethics and limited sourcing are still important barriers to consider. This approach has proven effective, but the ideal therapy, would be to simply replace the dying photoreceptors themselves. This treatment would be ubiquitous, and completely independent of the underlying pathology or injury. Recent developments have provided a strong framework in which we can successfully induce retinal cells from pluripotent stem cells. Next steps should involve further exploration of the nuances of the degenerative environment in order to combine cell replacement therapies with drugs or molecules that can improve the environment and facilitate integration, though integration will always remain a limiting factor to this form of treatment. The most promising advent, however, is the elucidation of mechanisms that induce retinal regeneration. A regenerating retina will not face graft rejection, and because the cells emanate from the retina itself, there are no challenges to integration. However, understanding how this regenerative capacity may be affected by pathological insult still remains a key component needed to make the advancement toward therapeutics. Changes to cellular plasticity and increasing ECM rigidity may negatively impact the retina’s ability to regenerate. Furthermore, in the case of RP and AMD, where most photoreceptor loss is late onset, newly regenerated PRs must sustain and function in a harsher microenvironment than the first iteration of cells, so perhaps in addition to induction, patients may have to consistently supplement, to maintain retinal health. In the antithetical instance, where degeneration happens early in life, other options have to be contemplated. A younger retina could be more amenable to regeneration, but will gene therapies have to be delivered in conjunction with regenerative cues? Considering the vast number of acquired and inherited retinal diseases, cell replacement therapy would be the most ubiquitous and efficacious therapy. The advancements discussed here suggest a viable future, should the challenges of integration and regeneration and its remaining complexities be met.

## Author Contributions

First draft was written by LI and edited by MA-U and MN. All authors contributed to the manuscript and approved the submitted version.

## Conflict of Interest

The authors declare that the research was conducted in the absence of any commercial or financial relationships that could be construed as a potential conflict of interest.
